# Stimulated Raman scattering microscopy: an emerging tool for drug discovery

**DOI:** 10.1039/c5cs00693g

**Published:** 2016-02-03

**Authors:** W. J. Tipping, M. Lee, A. Serrels, V. G. Brunton, A. N. Hulme

**Affiliations:** a EaStCHEM School of Chemistry , The University of Edinburgh , Joseph Black Building , David Brewster Road , Edinburgh , EH9 3FJ , UK . Email: Alison.Hulme@ed.ac.uk; b Edinburgh Cancer Research Centre , Institute of Genetics and Molecular Medicine , The University of Edinburgh , Crewe Road South , Edinburgh , EH4 2XR , UK

## Abstract

Optical microscopy techniques have emerged as a cornerstone of biomedical research, capable of probing the cellular functions of a vast range of substrates, whilst being minimally invasive to the cells or tissues of interest. Incorporating biological imaging into the early stages of the drug discovery process can provide invaluable information about drug activity within complex disease models. Spontaneous Raman spectroscopy has been widely used as a platform for the study of cells and their components based on chemical composition; but slow acquisition rates, poor resolution and a lack of sensitivity have hampered further development. A new generation of stimulated Raman techniques is emerging which allows the imaging of cells, tissues and organisms at faster acquisition speeds, and with greater resolution and sensitivity than previously possible. This review focuses on the development of stimulated Raman scattering (SRS), and covers the use of bioorthogonal tags to enhance sample detection, and recent applications of both spontaneous Raman and SRS as novel imaging platforms to facilitate the drug discovery process.

## Abstract 2

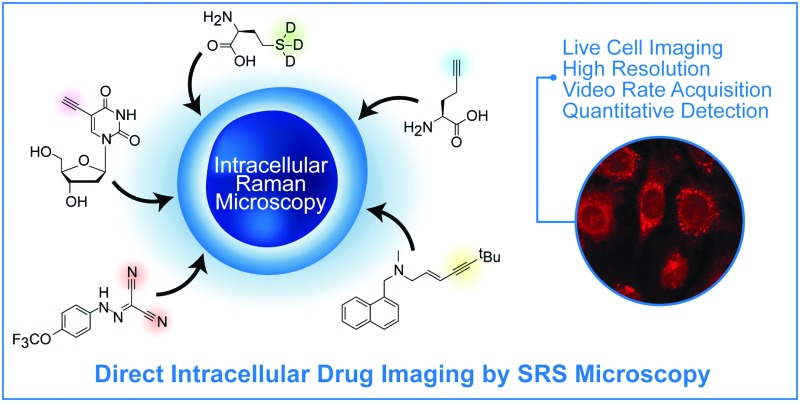
Stimulated Raman scattering and the use of bioorthogonal tags provide novel imaging platforms to facilitate the drug discovery process.

## 1. Introduction

The high attrition rates of candidate drugs during clinical development remains a common feature within the pharmaceutical industry.^1–6^ The industry has seen a decline in productivity, which has been attributed to several factors including heightened regulatory scrutiny,^7^ toxicity concerns^8–10^ and a lack of clinical efficacy.^2,10^ High attrition rates may also be attributed to the lack of dynamic readouts of drug activity in an *in vivo* setting;^11^ therefore, innovative approaches to drug screening are needed. Incorporating imaging into complex drug screening models has the potential to improve the robustness of preclinical studies of drug uptake, retention and metabolism. The insight gained from such studies could enable earlier removal of ill-fated compounds from the development cycle, with consequential savings in financial investment, and improve the quality of the drug development pipeline.

The analysis of a cell, or tissue, in a native environment still poses a significant challenge to biomedical research. Fluorescence microscopy has helped to address this, permitting the real-time imaging of fluorescently labelled molecules, including proteins, antibodies and small molecules such as drugs or their metabolites. However, fluorescent labels are often large relative to the size of a small molecule, and can thus greatly perturb the study of its intracellular properties. Intrinsic molecular contrast which does not rely on the incorporation of bulky fluorescent labels or dyes would be highly advantageous to the analysis of small molecules in a cellular environment by imaging; and the development of techniques based on intrinsic molecular contrast could allow more complex imaging models to be incorporated into the drug discovery process.

Existing label free optical technologies, such as optical coherence tomography (OCT), do not offer chemical contrast. However, vibrational spectroscopies including infrared (IR) and Raman spectroscopy provide potential methods for species-specific label-free visualisation, as they generate contrast based on chemical composition. Imaging systems based upon infrared spectroscopy have been reported,^12^ but they are limited by the low spatial resolution imposed by the diffraction limit of infrared light, and the strong IR absorption cross-section of water and aqueous media. Raman scattering techniques offer alternative methods for cellular imaging, with the potential to resolve some of the shortcomings associated with IR imaging. Crucially, water has a very weak Raman scattering cross-section and therefore molecules and cells can be studied in aqueous environments.

Technological advances and instrumental developments associated with Raman based microscopies have been covered recently by Krafft *et al.* ^13^ Here, we provide an overview of recent advances in stimulated Raman scattering (SRS) microscopy with a particular focus on developments relevant for the drug discovery process. We discuss current approaches to label-free and near-native labelling of cellular components and small molecules of medicinal interest for Raman imaging. We highlight Raman tagging strategies which exploit the “silent region” in the Raman spectra of cells; these have allowed the development of spectroscopically bioorthogonal tags which maximise signal contrast. Finally, we conclude with case studies of recent applications of Raman based microscopies, with particular focus on SRS, and the potential of these approaches to enhance early stage drug discovery programmes.

## 2. Raman microscopy

### 2.1 The Raman scattering process

**Fig. 1:**
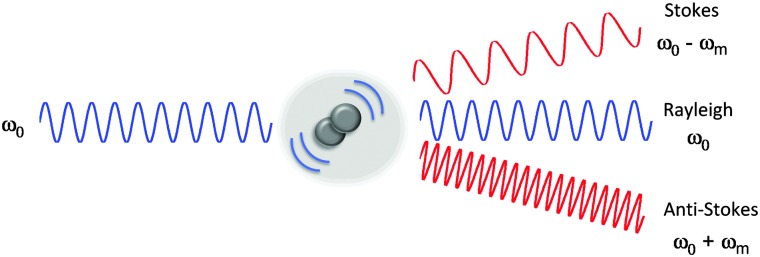
Electromagnetic radiation interacting with a vibrating molecule. When incident radiation (*ω*_0_) interacts with a chemical species, it can be elastically scattered (Rayleigh scattering) or inelastically scattered (Raman scattering) by an amount, *ω*_m_ which corresponds to the energy of a molecular transition in the molecule. In the case of Raman scattering, the scattered photon may have a lower energy compared with the incoming photon (Stokes scattering, *ω*_0_ – *ω*_m_), alternatively the scattered photon may have a higher energy than the incident photon (anti-Stokes scattering, *ω*_0_ + *ω*_m_).

The Raman scattering effect was first observed in 1928 by C. V. Raman, for which he received the Nobel Prize in Physics in 1930. Raman spectroscopy has since been used to probe the chemical nature of a vast range of substrates, by the detection of specific chemical bonds inherent to the structure under investigation. Raman, and its more commonly encountered partner infrared (IR) are complementary vibrational spectroscopic techniques: Raman scattering probes changes in the polarizability of a vibrating molecule; whilst IR absorption accompanies changes in the dipole moment of the molecule under investigation. In Raman spectroscopy, the majority of incident radiation does not couple with a vibrational excitation, and the induced polarization does not change the vibrational state the molecule started in; thus most scattered photons have the same energy as the incident photon (Rayleigh scattering, Fig. 1). However, when the incident radiation induces a polarisation that couples with a vibrational change in the molecule, the photon is scattered inelastically (Raman scattering, Fig. 1). For a molecular vibration to generate a Raman band, there must be an associated change in the polarizability of the chemical bond, such that there is a corresponding distortion of electron density in the vicinity of the vibrating nuclei. Thus vibrations of multiply-bonded or electron-rich groups, C≡C, C≡N and C=O for example, produce more intense Raman bands than do vibrations of singly-bonded or electron-deficient groups.

**Fig. 2:**
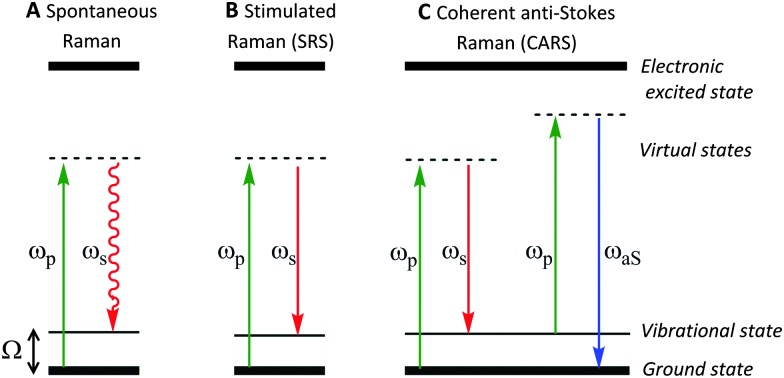
Schematic energy level diagrams for spontaneous Raman, stimulated Raman scattering (SRS) and coherent anti-Stokes Raman scattering (CARS) processes. (A) In spontaneous Raman, the pump beam, *ω*_p_, is directed on to the sample generating a red-shifted signal *ω*_s_, due to inelastic scattering. (B) During SRS two laser beams at frequencies *ω*_p_ and *ω*_S_ are incident upon the sample, such that when the frequency difference (Δ*ω* = *ω*_p_ – *ω*_S_) matches a molecular vibration in the sample (*Ω*), stimulated emission occurs. (C) CARS microscopy is a complex advanced Raman imaging technique, involving a four-beam mixing process probing at the anti-Stokes frequency (*ω*_aS_).

In spontaneous Raman spectroscopy (Fig. 2A), inelastic scattering of the incident (pump) beam is detected as either red-shifted (lower energy) or blue-shifted (higher energy) radiation and is typically reported in wavenumbers (cm^–1^). The energy processes which occur in stimulated Raman scattering (SRS) and coherent anti-Stokes Raman scattering (CARS) experiments are provided in Fig. 2 for comparison, whilst further discussion of these techniques can be found in Section 2.3.

### 2.2 Spontaneous Raman microscopy

**Fig. 3:**
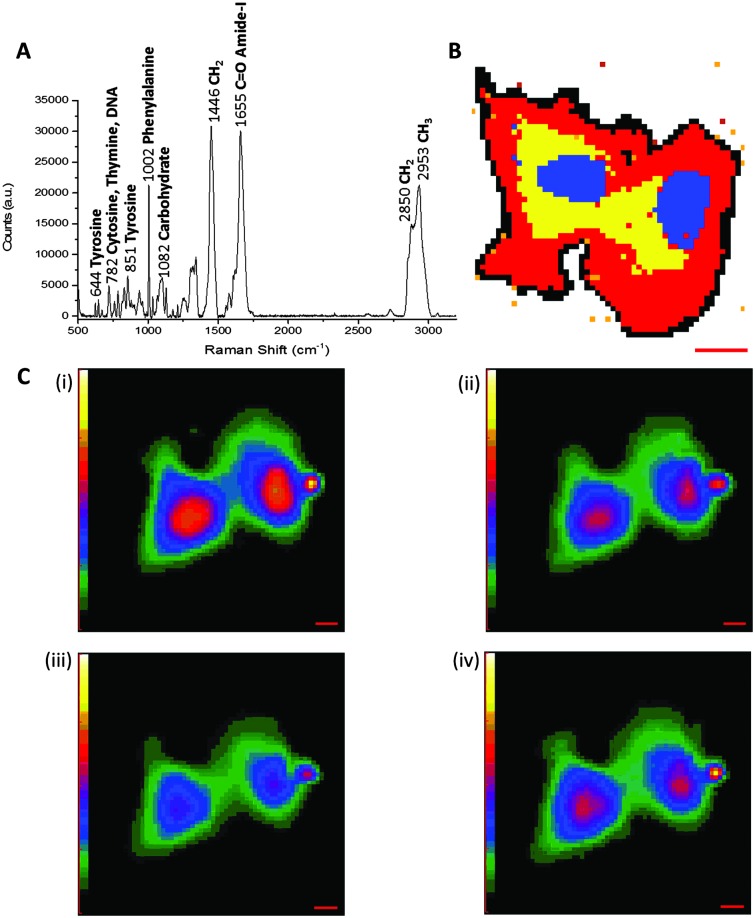
Label-free imaging of SKBr3 cells by Raman microscopy. (A) Spontaneous Raman spectrum of SKBr3 cell pellet measured using 785 nm excitation. Key bands are indicted, with wavenumbers measured in cm^–1^. (B) Spontaneous Raman image of two fixed SKBr3 cells, in the region 2100–3100 cm^–1^, with principal component analysis to identify different regions of the cell. Blue: principal component 1, yellow: principal component 2, red: principal component 3, black: principal component 4. Scale bar 10 μm. (C) Molecular distribution maps of fixed SKBr3 cells by Raman microscopy, (i) extracted image at 1033 cm^–1^ (C–H in-plane of phenylalanine); (ii) extracted image at 1657 cm^–1^ (C=O, amide-I); (iii) extracted image at 2880 cm^–1^ (CH_2_, lipid); and (iv) extracted image at 2939 cm^–1^ (CH_3_). Scale bars 5 μm.

Spontaneous Raman spectroscopy has seen increased usage as an imaging platform for the analysis of cells and their contents since pioneering work by Puppels and co-workers in 1990 on Raman spectroscopic measurements of living cells and chromosomes.^14^ Raman scattering is typically a non-destructive technique, employing relatively low energy laser irradiation, and there is minimal interference from water in aqueous samples. Therefore, Raman spectroscopy is suitable for live cell imaging. Fig. 3A shows a representative cellular Raman spectrum of pelleted SKBr3 cells, with assignments for key bands. Strikingly, there is no contribution from cellular components in the region 1800–2800 cm^–1^.

Acquisition of a Raman spectrum is achieved *via* excitation with one fixed laser wavelength, which simultaneously induces the inelastic scattering of all the vibrational Raman active modes in the sample. In this way, interfacing Raman spectroscopy to cellular imaging is easily achieved by first measuring the Raman spectrum at each pixel, and determining the intensity of a Raman peak of interest at each pixel to create a two-dimensional (2D) species-specific location map. Using a chemometric technique such as principal component analysis (PCA), it is possible to obtain information about molecular structure and the spatial distribution of the cellular components (Fig. 3B). Similarly, molecular distribution maps of proteins (1033 cm^–1^ & 1655 cm^–1^) and lipidic species (2880 cm^–1^) can be created by plotting the signal intensity of a peak of interest at each pixel (Fig. 3C). A three-dimensional (3D) Raman image is constructed by scanning the Raman probe laser through the sample point-by-point with a laser scanning microscope. Applying dimensionality reduction algorithms, such as clustering analysis, to the spectral data allows the image to be segmented. In this way, Raman microscopy can be used to generate images scaled from microscopic sub-cellular regions of interest to macroscopic areas of tissue. Spontaneous Raman spectroscopy has generated much interest in the medical diagnostic field, because it has been shown that the spectral data generated can be used to distinguish between malignant and normal cells and tissues based on spectral characteristics. A review of the current state of clinical applications of Raman microscopy has been collated by Wang *et al.* ^15^

To minimise late-stage drug failure there is a pressing need to improve the early stage drug discovery processes; preclinical modelling should aim to better mimic, and thereby predict, the clinical response in the intended target tissue environment.^16–20^ This is difficult to achieve using fluorescent optical technologies, as a fluorophore is often large relative to the small molecule under investigation and hence perturbs its activity. Raman spectroscopy has been used to map the interactions of small molecules with cells;^21–23^ but a major challenge in extending the methodology employed in the study of proteins and lipids within cellular and tissue samples, to the study of drug molecules, is that these small molecules typically accumulate at significantly lower intracellular concentrations. To date, relatively high analyte concentrations have been required to enable their detection above the cellular background signal, which may preclude the analysis of drugs at physiologically relevant concentrations using spontaneous Raman spectroscopy. Furthermore, the spontaneous Raman scattering cross section is extremely small (∼10^–30^ cm^2^ sr^–1^) compared to fluorescence (∼10^–16^ cm^2^ sr^–1^), and as such, limits the speed of acquisition of biological images by Raman spectroscopy.^24,25^ These limitations in spontaneous Raman spectroscopy have prompted the development of more advanced techniques.

### 2.3 Stimulated Raman scattering microscopy

Coherent Raman scattering microscopy techniques, based on either coherent anti-Stokes Raman scattering (CARS) or stimulated Raman scattering (SRS), have been developed to overcome the low signal levels associated with spontaneous Raman imaging, and hence enhance real-time vibrational imaging of living cells and organisms. SRS was first reported in 1962,^26^ although it is only recently that the potential of SRS as an alternative Raman imaging platform has been realised. Table 1 highlights some of the key advantages of SRS over its more widely-known counterpart CARS.

**Table 1:** Comparison between CARS and SRS imaging techniques

CARS	SRS
CARS signal generated at new optical frequency (*ω*_aS_)	Intensity gain (SRG) and intensity loss (SRL) in incident beams
Non-resonant background artefacts	No non-resonant background present
Non-linear concentration dependence	Linear concentration dependence
Spectral distortion can occur	Spectra match spontaneous Raman spectrum

**Fig. 4:**
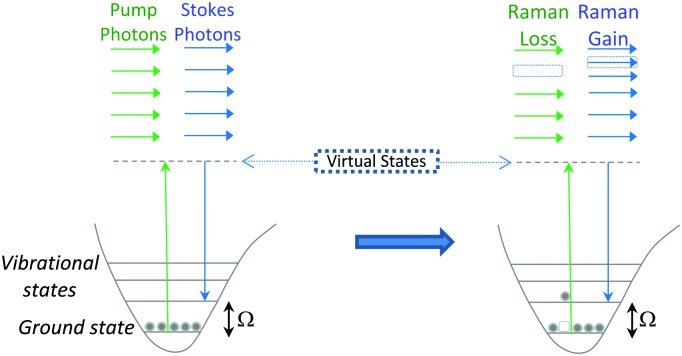
Output spectrum associated with SRS process. The Stokes beam experiences an intensity gain resulting from the stimulated excitation of a Raman active band within the target molecule (SRG). Conversely, the pump beam experiences an intensity loss during this process (SRL).

SRS signals are generated by the co-alignment of two incident beams (the pump and Stokes beams). By tuning the frequency difference (Δ*ω* = *ω*_p_ – *ω*_s_) between the beams to match a molecular vibration (*Ω*), stimulated excitation of the Raman active molecular vibration occurs (see Fig. 2B). This process causes an intensity loss in the pump beam (stimulated Raman loss, SRL) and an intensity gain in the Stokes beam (stimulated Raman gain, SRG). By modulating one of the beams (typically, the Stokes beam) using either an electro-optic modulator (EOM), or an acousto-optic modulator (AOM), the change in pump beam can be measured using radio-frequency lock-in detection providing a contrast to generate an image (Fig. 4).^27^

Conversely, when Δ*ω* does not match a molecular vibration within the sample, SRL and SRG cannot occur. As a result, there is no associated non-resonant background, rendering image analysis much simpler than in CARS imaging. In comparison to SRS, during the CARS process, no energy is transferred to the sample, rather, the energy difference between the pump- and Stokes-photons is emitted at the anti-Stokes frequency (see Fig. 2B).^28^ Consequently, CARS processes can occur when there are no resonant molecules in the field of view, giving rise to non-resonant background effects. This background can limit the detection sensitivity and also result in a distortion of the CARS spectra from the spontaneous Raman spectrum.^28^ However, CARS processes are typically more intense than SRS signals, and CARS microscopy has been successfully applied to the direct visualisation of cells and tumour micro-environments.^29,30^

**Fig. 5:**
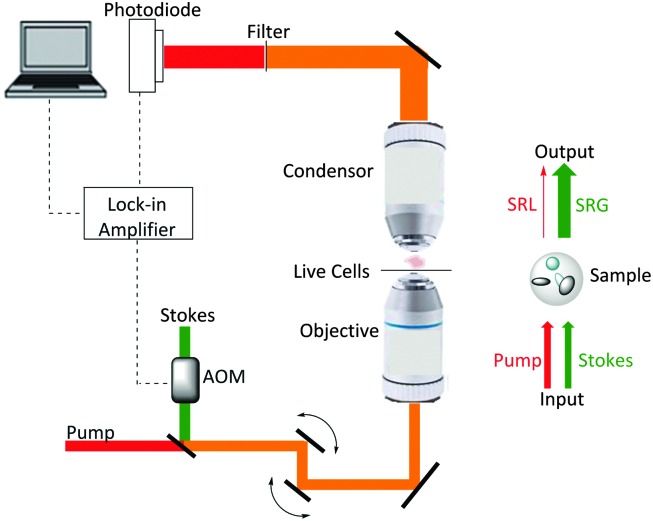
A schematic representation of a SRS microscope. A pump beam and an intensity-modulated Stokes beam are both temporally and spatially synchronized before being focused onto cells. When the energy difference between the pump photons and the Stokes photons matches the molecular vibration (*Ω*) of a bond, those particular bonds are efficiently driven from their vibrational ground state to their vibrational excited state, passing through a virtual state. For each excited bond, a photon in the pump beam is annihilated (Raman loss) and a photon in the Stokes beam is created (Raman gain). These intensity changes in the pump beam are extracted using a lock-in amplifier and can provide a quantitative map of the targeted vibrating chemical bonds.

**Fig. 6:**
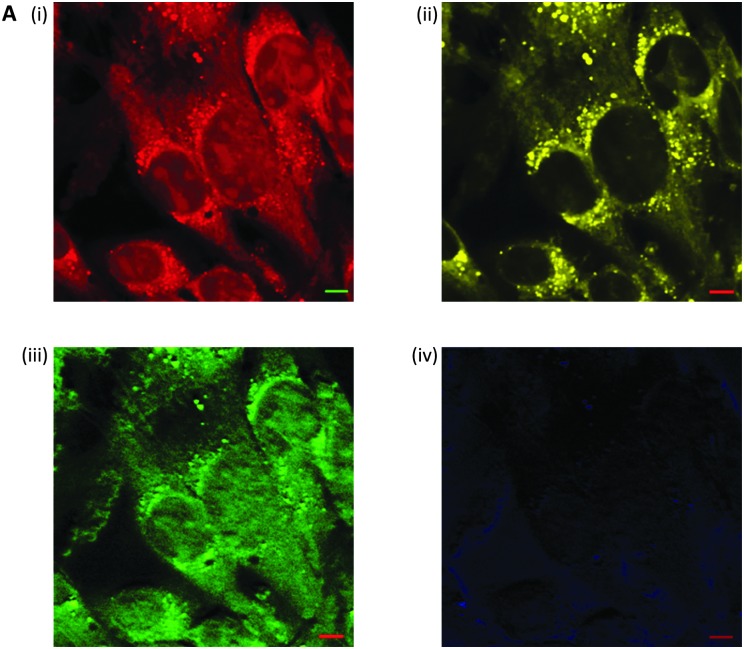
Label free microscopy of squamous cell carcinoma cells by stimulated Raman scattering microscopy. (A) Stimulated Raman scattering image of fixed SCC cells, (i) acquired at 2953 cm^–1^ (CH_3_); (ii) acquired at 2850 cm^–1^ (CH_2_, lipid); (iii) acquired at 1655 cm^–1^ (C=O, amide-1); and (iv) acquired at 1700 cm^–1^ (cellular silent region). Scale bars 5 μm.

Ploetz *et al.* described the first femtosecond stimulated Raman scattering microscope in 2007.^31^ Fig. 5 provides a schematic overview of a SRS microscope, central to which is the synchronised pairing of the pump and Stokes beams. These beams are spatially overlapped *via* a dichroic mirror and co-lineated into a beam-scanning microscope. An objective lens focuses the beams onto a common focal spot within the target sample which generates a stimulated Raman process at its focus. A high numerical aperture condenser collects the transmitted beams, with a filter blocking the Stokes beam and subsequent detection of the pump beam is achieved *via* a photo-diode. A lock-in amplifier is used to extract the SRL signal from the laser intensity, providing a Raman intensity value at each pixel (Fig. 6).

Three-dimensional imaging is achieved by raster scanning the laser focus across the sample and moving the focus depth into the sample. Separating the frequency of the two laser pulses by the vibrational frequency of a molecular transition (*Ω*) in the target sample, results in only a single specific vibrational level in the sample being probed.

SRS systems with picosecond pulses are ideal for probing single Raman vibrations as the Raman line width is typically around 10 cm^–1^, comparable to the spectral width of a several picosecond pulse.^32^ In the presence of several overlapping Raman bands from different species, more spectral information may be required to aid separation and identification of the component vibrations. Hyperspectral stimulated Raman scattering (hsSRS) with picosecond sources can be achieved by tuning the pump or Stokes laser through the desired wavelength range^33,34^ although this can be a time consuming process often taking several minutes and may be susceptible to optical power drift and wavelength drift. Faster methods have been developed using femtosecond lasers, where spectrally broad pulses can be modulated using pulse shapers, spectral focusing, or spectral filtering to achieve rapid scanning of differing frequencies.^35–37^

To further suppress background signals, spectrally modulated SRS has been developed by Zhang *et al.* ^38^ Two spectrally narrow bands are cut from a broadband femtosecond pulse, probing on- and off-resonance pulses, and switched at high frequencies. Intensity modulation of a single beam generates other heterodyne contrast such as photothermal lensing, cross-phase modulation and transient absorption. By spectrally modulating the beam only the Raman effect is detected, which can be vital in pigmented cells.^39^ Spectrally tailoring the excitation to modulate between a target species and interfering species can also be used, when correctly weighted, to cancel out the interfering species Raman contribution.^40^

For thick and opaque samples, such as tissue, where up to 45% of the incident photons may be back scattered, *epi*-detection of the SRS signals can be required.^41^ Back scattered light, which has scrambled phase and polarisation, can be collected by the objective lens and separated from the incident light using a polarising beam splitter.^42,43^ However, a more efficient method has been demonstrated by Ito *et al.* where a large area photodiode is placed between the objective lens and the sample to collect the majority of back scattered photons which fall outside of the objective lens.^44^ An alternative approach to improving image resolution in SRS microscopy has recently been reported which combines stimulated Raman scattering microscopy with tip enhanced Raman spectroscopy (TERS) imaging.^45^

These developments mean that SRS images can now be generated across a range of cell and tissue environments, giving high resolution data that is appropriate for use in preclinical applications (see Section 4). Limitations arising from the Raman-based detection of the drug molecule itself in the intracellular environment have been addressed using a number of strategies as outlined in Section 3.

## 3. Enhanced detection in SRS

Strategies aimed at enhancing the visualisation and quantitation of biomolecules by Raman microscopy have focused not only on the use of more powerful Raman scattering techniques such as SRS, but also on the introduction of small Raman-active tags to enhance detectability. These tagging strategies frequently exploit the cellular silent region of the Raman spectrum (*i.e.* they are spectroscopically bioorthogonal), where there is minimal background interference from intrinsic cellular components.^46^ Two main strategies are employed in the design of Raman-active tags: the first is the use of bioorthogonal Raman-active groups which are either inherent to the molecule under investigation, or are selectively introduced for imaging purposes. The second is the use of isotopologues (*i.e.* compounds which differ from the parent structure only by an isotopic substitution), which again can be selected to give Raman-active bands in the cellular silent region. Table 2 summarises some of the key Raman-active tags that have been used for cell-based imaging.

**Table 2:** Raman-active probes

Entry	Raman probe	Wavenumber (cm^–1^)	Application	
			Spontaneous Raman imaging	SRS imaging
1	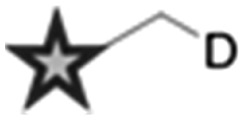	∼2120	Uptake of deuteriated drug carriers. Ref. 47	Deuteriated choline metabolism. Ref. 48
2	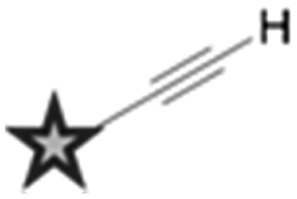	∼2110	Intracellular localisation of erlotinib. Ref. 49	Intracellular localisation of EdU. Ref. 50
3	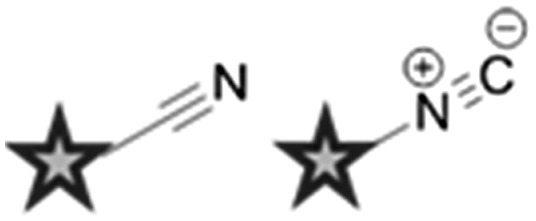	∼2120–2240	Intracellular localisation of FCCP. Ref. 21	Intracellular localisation of rhabduscin. Ref. 51
4	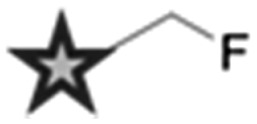	∼688		Perfluorinated agrochemical uptake in plant leaves. Ref. 52
5	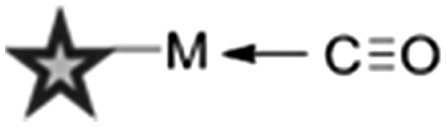	∼1900–2050^*a*^	Cellular localisation of Re and Mn based therapeutics. Ref. 53 and 54	
6	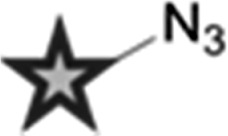	∼2096		

^*a*^The wavenumber will invariably change with coordination of the ligand to different metal centres with different electronic configurations and different coordination spheres, as indicated in the two referenced examples.

### 3.1 Bioorthogonal Raman tags for imaging

**Fig. 7:**
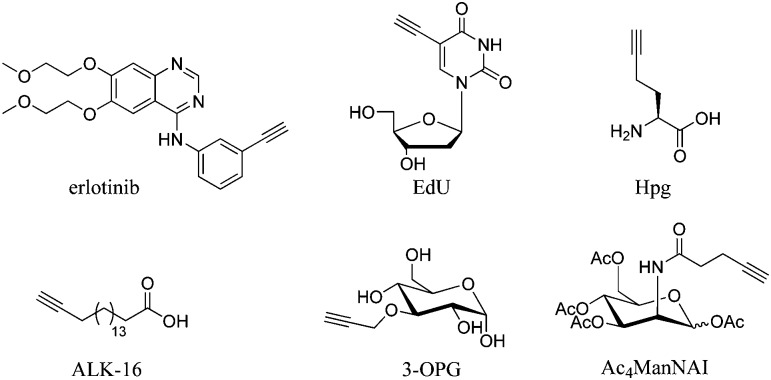
Structures of molecules containing alkyne groups, which act as bioorthogonal markers for spontaneous Raman and SRS imaging. Erlotinib is a clinical drug which has an inherent alkyne bond, EdU is a thymidine analogue modified with an alkyne bond, Hpg is an alkyne containing methionine analogue, ALK-16 is a palmitic acid analogue modified with an alkyne group, 3-OPG is a glucose analogue modified with a propargyl group and Ac_4_ManNAI is an alkyne-containing glycan analogue.

Cell imaging using inherent Raman bands, *e.g.* CH_2_ bands (indicative of lipids and fatty acids) and the amide-I C=O band (indicative of proteins), has enabled the intracellular mapping of these species. However, imaging the uptake of drugs and drug carriers presents a significant challenge using conventional Raman microscopy, since these frequently accumulate at much lower intracellular concentrations. Thus, spectroscopically bioorthogonal Raman-active tags have been adopted which act as markers for the small molecule of interest and improve detection sensitivity. Spectroscopically bioorthogonal Raman tags share many features in common with the bioorthogonal groups used in click chemistry applications, since in both cases the groups must be inert towards cellular decomposition (*e.g.* by water) and reactivity (*e.g.* with cellular nucleophiles); the most widely studied spectroscopically bioorthogonal group is the alkyne moiety. These Raman tags may be inherent to the structure under investigation (*e.g.* the alkyne functional groups present in erlotinib and EdU, Fig. 7), or alternatively they may be incorporated through modification of the parent structure. In general, they are much smaller than fluorescent tags and show greater photostability than their fluorescent counterparts (*i.e.* the intensity of the Raman signal does not decay with prolonged exposure).

**Fig. 8:**
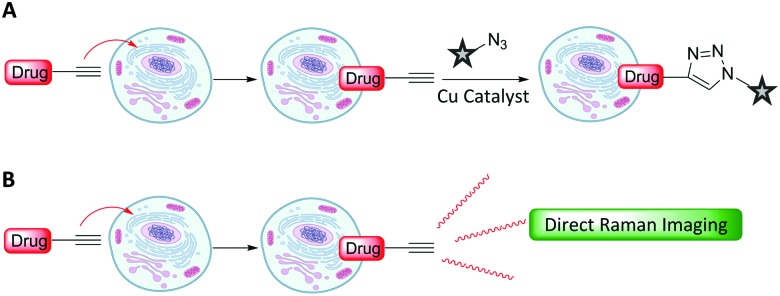
Strategies for intracellular visualisation of biomolecules. (A) Bioorthogonal ligation strategy illustrated using a CuAAC click reaction. (B) Bioorthogonal Raman-active tagging strategy. A small Raman reporter installed onto, or inherent to, the biomolecule of choice is used as a marker to perform direct spontaneous Raman or SRS imaging.

Metabolic incorporation of the thymidine analogue EdU into DNA, followed by CuAAC click reaction staining of the bioorthogonal alkyne of EdU with fluorescent azides, has emerged as a powerful means of visualising DNA synthesis.^55–57^ This bioorthogonal click chemistry approach to labelling is attractive because it is possible to chemically label species with high specificity using a range of ligation techniques (Fig. 8A).^58^ However, incorporation of a bioorthogonal Raman-active tag within a drug, or drug delivery vehicle, could potentially enable direct visualisation by Raman microscopy (Fig. 8B), negating the requirement for subsequent bioorthogonal chemical reactions.

**Fig. 9:**
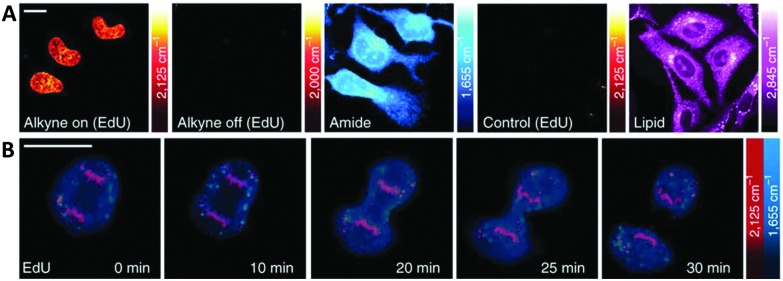
Live SRS imaging of *de novo* synthesis of DNA. (A) Live HeLa cells incubated with EdU alone (100 μM) alkyne on; and with hydroxyurea (10 mM) control. (B) Time-lapse images of a dividing cell incubated with EdU (100 μM). Scale bars: 10 μm. Adapted from ref. 60 with permission. Copyright (2014) Nature Publishing group.

Alkyne-tag Raman imaging (ATRI) was proposed in 2011 by Yamakoshi *et al.* as a practical means to image cellular processes, utilising a small alkyne tag to provide chemical contrast in the spontaneous Raman image.^46,59^ Real-time visualisation of DNA synthesis in living cells, without prior fixing or staining protocols, has been achieved using both spontaneous Raman and SRS imaging of the alkyne band of EdU.^46,50,59^ Hong *et al.* showed that HeLa cells treated with EdU showed a significant SRS signal (at 2120 cm^–1^) due to the alkyne in the nucleus.^50^ This spectroscopically bioorthogonal signal was abolished by treatment with hydroxyurea (a DNA synthesis inhibitor), indicating that it did indeed result from metabolic incorporation of EdU during DNA synthesis. Min and co-workers showed that the limit of detection of intracellular EdU using SRS was 200 μM.^60^ Tracking dividing cells every 5 minutes during mitosis neatly demonstrated the acquisition speed, resolution and live cell dynamics of SRS imaging (Fig. 9).^60^

Whilst the metabolic incorporation of EdU into DNA is the most studied example of ATRI, other alkyne-tagged small molecules which have been incorporated metabolically include: (i) homopropargylglycine^50^ (Hpg, Fig. 7), a methionine analogue with an SRS signal at 2120 cm^–1^, which can be incorporated in protein synthesis in methionine auxotrophic species; (ii) 17-octadiynoic acid^50^ (ALK-16, Fig. 7), an alkyne-containing palmitoyl acid analogue with an SRS signal at 2120 cm^–1^, which allows the distribution of proteins lipidated with this particular fatty acid (the so-called palmitoylome) to be tracked in live cells; (iii) 3-*O*-propargyl-d-glucose^61^ (3-OPG, Fig. 7), an alkyne containing glucose analogue, which enabled monitoring of glucose uptake and activity in living cells and tissues using the SRS signal at 2129 cm^–1^ and (iv) the monosaccharide precursor peracetylated *N*-(4-pentynoyl)mannosamine (Ac_4_ManNAl, Fig. 7), which is metabolised to the corresponding sialic acid derivative and incorporated into sialyl-glycans imaged at 2120 cm^–1^.^50^

**Fig. 10:**
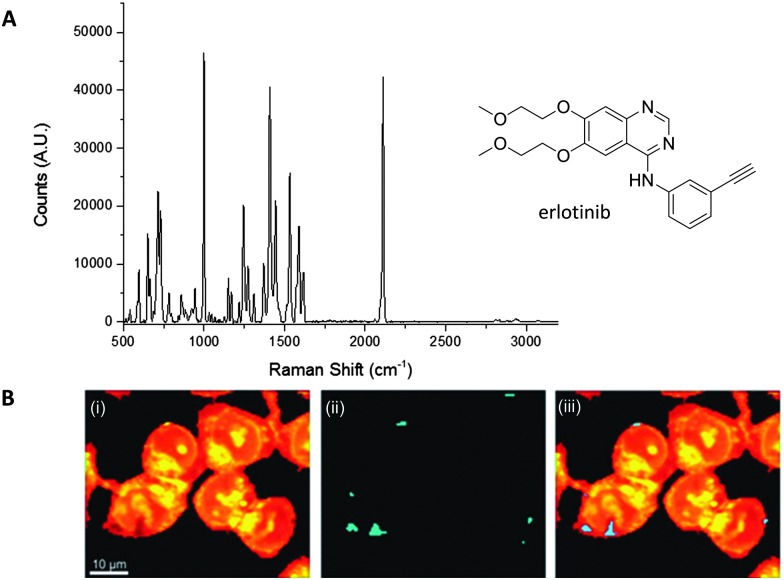
Spontaneous Raman imaging of erlotinib. (A) Representative spontaneous Raman spectrum of erlotinib showing clearly the strong alkyne band at 2110 cm^–1^. Spectrum acquired at 785 nm excitation. (B) Spontaneous Raman imaging of SW480 cells treated with erlotinib (∼100 μM, 12 h) (i) spontaneous Raman image reconstructed from the C–H stretching intensity; (ii) spontaneous Raman image constructed from the C≡C stretching intensity; (iii) overlay of panels (i) and (ii) showing the drug is clustered at the EGFR protein at the cell membrane. Adapted from ref. 49 with permission. Copyright (2014) Royal Society of Chemistry publishing.

El-Mashtoly *et al.* have imaged the distribution of the epidermal growth factor receptor (EGFR) inhibitor, erlotinib (Fig. 10); which is a frontline therapy used in the treatment of non-small cell lung cancer.^49^ Erlotinib has a conjugated aryl alkyne moiety, which gives rise to a comparatively intense Raman signal. The group demonstrated that changes to the spontaneous Raman cellular spectrum of erlotinib, as compared to free erlotinib, indicated that intracellular metabolism gave the desmethyl derivative. However, the concentrations of erlotinib and its desmethyl metabolite detected by HPLC and LC-MS analysis (of human plasma from patients following the recommended 150 mg daily doses) are 3–5 μM and 0.3–0.7 μM respectively.^62,63^ At present, it is not possible to detect such low concentrations using spontaneous Raman spectroscopy, suggesting further improvements are required before this method is clinically relevant.^49^

Aryl-conjugated alkynes have also been used in the spontaneous Raman imaging of mitochondria in live cells using lipophilic cationic phosphonium derivatives based on bisarylbutadiyne (BADY).^64^ The use of the bisarylbutadiyne motif (imaged at 2220 cm^–1^) in these compounds generates mitochondrial imaging agents with signals 27 times more intense than that of EdU. Similar butadiyne motifs have been incorporated into cholesterol^65^ and sphingomyelin.^66^

Other alternative markers for spectroscopically bioorthogonal Raman imaging include azides, nitriles and inorganic metal–carbonyl complexes. Perhaps surprisingly, although both the alkyne and azide units which participate in the CuAAC click reaction have diagnostic peaks which fall into the cellular silent region of the Raman spectrum, and may therefore represent smaller tags for Raman spectroscopy,^46^ the azide group has yet to be used in this context. This is most probably due to the reduced polarizability of the azide bond which gives rise to a lower observed Raman scattering intensity.

**Fig. 11:**
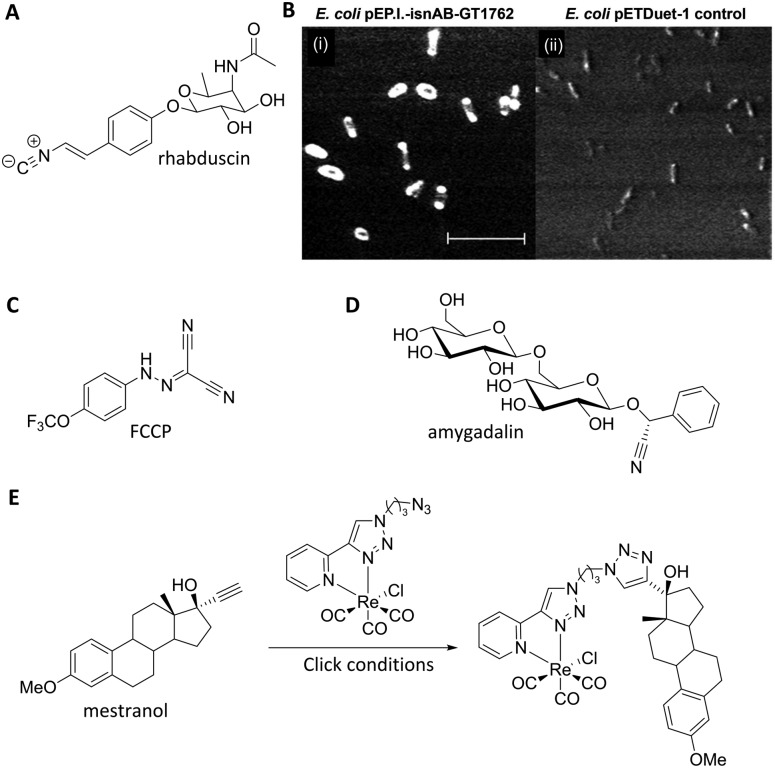
Recent examples of biomolecules studied by SRS and spontaneous Raman imaging. (A) Rhabduscin. (B) SRS microscopy analysing the spatial localisation of rhabduscin based on the vibrational resonance of the isonitrile functional group at 2121 cm^–1^. (i) Rhabduscin is localised at the periphery of *E. coli* cells heterologously expressing the rhabduscin pathway; (ii) the background signal is low in the cells lacking the rhabduscin gene cluster. Scale bar 10 μm. Adapted from ref. 51 with permission from the National Academy of Sciences. (C) FCCP (D) amygadalin (E) mestranol and its conjugation to an inorganic coordination complex *via* a CuAAC click reaction.

Nitriles are also attractive markers for Raman microscopy because of their widespread usage in medicinal chemistry as a carbonyl bioisostere,^67^ with potential for visualisation of the un-modified drug molecule. Crawford *et al.* demonstrated SRS microscopy of rhabduscin (Fig. 11A), a tyrosine-derived amidoglycosyl- and vinyl-isonitrile product in *E. coli* using the isonitrile functional group as an intrinsic Raman tag.^51^ Genetically transformed *E. coli* which heterologously expressed the rhabduscin pathway, showed significant rhabduscin localisation in the cell periphery (Fig. 11B), whilst *E. coli* lacking the rhabduscin gene cluster showed minimal signal from the isonitrile functional group probed at 2121 cm^–1^ (Fig. 11B). Yamakoshi reported the simultaneous imaging of the protonated and deprotonated forms of carbonylcyanide *p*-trifluoromethoxy-phenylhydrazone (FCCP) by spontaneous Raman microscopy, in which the nitrile moiety serves as an intrinsic Raman tag (Fig. 11C).^21^

Nitrile tags have also been used to label lysine residues within bovine serum albumin (BSA), using the NHS ester of *p*-cyano-benzoic acid to install the nitrile tag.^68^ The modified protein was shown to retain its wild-type function, demonstrating the benefits of the small-sized Raman-active nitrile tag. Finally, Krafft has also demonstrated the subcellular distribution of amygdalin (Fig. 11D), a nitrile containing glycoside, in thick apricot seed sections by spontaneous Raman spectroscopy.^69^

Metal–carbonyl (M ← C≡O) ligands have also been used in spontaneous Raman microscopy studies to monitor the cellular uptake of inorganic complexes. Policar *et al.* investigated the cellular localisation of mestranol, an estrogen derivative bearing a terminal alkyne unit, through its CuAAC click reaction to a metal–carbonyl containing inorganic complex (Fig. 11D).^53^ The C≡O ligands of the rhenium complex used in this study displayed characteristic vibrational modes at 1915 cm^–1^ and 2032 cm^–1^. These Raman bands were used in confocal Raman microscopy to determine the cellular localisation of the conjugated mestranol in MDA-MB-231 breast cancer cells. Similarly, Meister *et al.* used the C≡O ligands of a manganese-based therapeutic as a bioorthogonal Raman marker for direct intracellular spontaneous Raman microscopy.^54^

### 3.2 Isotopologues as markers in Raman microscopy

The use of isotopologues has recently been demonstrated as an alternative Raman tagging strategy. Isotopologues are chemical compounds which are structurally identical but differ only in their isotopic composition. Deuterium (^2^H) and carbon-13 (^13^C) have been shown to be useful isotopic substitutions for Raman spectroscopy. The principle benefits of this strategy are that isotopologues have almost identical size and electronic characteristics to the parent compounds; but they may display favourable Raman spectral characteristics, particularly as C–D bands fall into the cellular silent region (∼2100 cm^–1^). Replacing the hydrogen atoms in a molecule with deuterium, may even improve metabolic stability, and the resultant deuteriated molecules are typically safe to handle and dose to patients.^70,71^

**Fig. 12:**
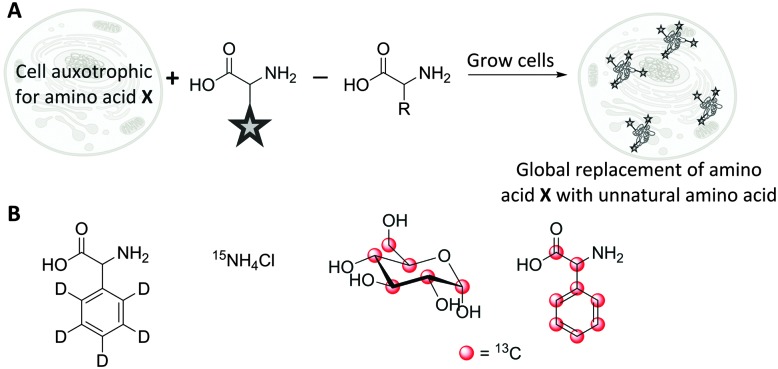
Incorporation of Raman-active isotopologues into proteins and biomolecules. (A) Metabolic incorporation of labelled amino acids in auxotrophic cell lines. (B) Raman-active metabolic precursors: deuteriated phenylalanine, ^15^N-labelled ammonium chloride, ^13^C-labelled glucose, ^13^C-labelled phenylalanine.

The detection of deuteriated proteins in living eukaryotic cells by spontaneous Raman spectroscopy was reported by van Manen *et al.* ^72^ Incubation of cells (Fig. 12A) with unlabelled phenylalanine and deuteriated phenylalanine enabled quantification of the incorporation of deuteriated amino acid by comparison of the labelled (959 cm^–1^) and unlabelled (1001 cm^–1^) Raman bands. A better signal to noise ratio was achieved by quantifying the ring breathing phenylalanine peak at ∼1000 cm^–1^, rather than the C–D peaks at ∼2100 cm^–1^. In contrast, quantification of tyrosine and methionine incorporation was best achieved using their C–D signals at 2100–2300 cm^–1^. Culturing cells with ^13^C-labelled glucose or ^15^N-labelled ammonium chloride (Fig. 12B) leads to metabolic incorporation of these heavy atoms in the growing cells. Shigeto *et al.* noted that for *S. pombe* cells cultured in a ^13^C-glucose (Fig. 12B) enriched medium, the subsequent incorporation of ^13^C could be detected by monitoring the shift of the phenylalanine ring breathing band from 1003 cm^–1^ to 967 cm^–1^.^73^ Shen *et al.* used a similar shift when employing fully labelled ^13^C-phenylalanine (Fig. 12B) to visualise protein degradation in living cells with subcellular resolution by SRS microscopy.^74^

Single cell Raman spectroscopy (SCRS) can be used to characterise heavy atom incorporation and provide information on different cell types, physiological states and phenotypes. Wang *et al.* have recently shown that technical advances mean that SCRS can even be used as a means of cell sorting.^75^ SRS microscopy in tandem with metabolic incorporation of deuteriated amino-acids has been used to visualise nascent proteins,^76^ enabling SRS studies into protein synthesis and degradation in live cells, brain tissue, zebrafish and live mice *in vivo*.^76,77^ These studies have indicated fast protein turn-over in nucleoli, which is in agreement with data obtained by fluorescence imaging using a click labelling strategy.^78^

**Fig. 13:**
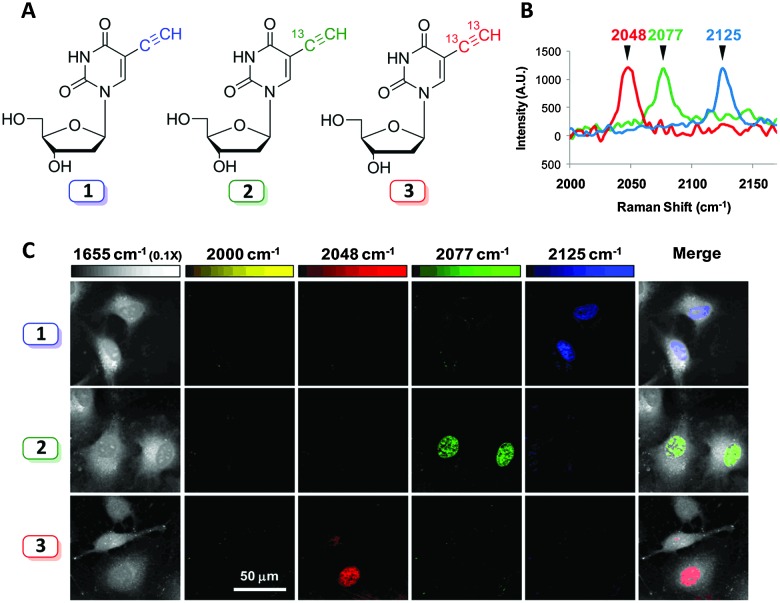
Development of a three colour palette of alkyne tags. (A) Structures of unlabelled, mono, and bis ^13^C-labelled 5-ethynyl-2′-deoxyuridine (EdU). (B) Spontaneous Raman spectra of HeLa cells incubated with the three isotopically edited EdU's (0.1 mM, 15 h). Spectra were acquired from the nuclear region of fixed cells following incubation with either 1, 2 or 3. The spectra were normalised at the alkyne peak, and are displayed from 2000–2170 cm^–1^. (C) Live cell SRS imaging of DNA synthesis in HeLa cells incubated with isotopically edited EdUs. For each sample incubated with either 1, 2, or 3. Images are acquired in 5 different Raman channels: 1655 cm^–1^ (amide I band), 2000 cm^–1^ (off-resonant), 2048 cm^–1^ (on-resonant with 3), 2077 cm^–1^ (on-resonant with 2), and 2125 cm^–1^ (on-resonant with 1) in sequential mode. Images are acquired in 512 × 512 pixels with a pixel dwell time of 40 μs. Image adapted from ref. 81 with permission. Copyright (2014) American Chemical Society.

Labelling strategies using ^13^C-substitution have also been applied successfully to study cell penetrating peptides,^79^ and single-walled carbon nanotubes conjugated to therapeutic agents,^80^ demonstrating the versatility of isotopic substitution in Raman microscopy. Isotopic substitutions have recently been used to extend alkyne tag chemistry to facilitate multiplexing experiments. Chen *et al.* developed a three-colour vibrational palette of alkyne tags based upon a ^12^C and ^13^C isotopic editing strategy, enabled by an elegant alkyne cross metathesis protocol (Fig. 13).^81^

Three alkyne isotopologues were prepared (the unlabelled ^12^C≡ ^12^C, mono-labelled ^13^C≡ ^12^C, and the bis-labelled ^13^C≡ ^13^C alkynes); these exhibited different stretching frequencies in their Raman spectra as a consequence of the difference in reduced mass, *μ*, for the ^12^C and ^13^C isotopes. With the isotopologues in hand, three colour chemical imaging of DNA synthesis was reported.^81^

## 4. Biomedical applications of SRS microscopy

Raman microscopy allows the intracellular concentrations of biomolecules to be imaged. This has sparked research into Raman imaging across a range of biomedical applications, from the study of drug uptake and distribution, to investigations into cellular metabolism and storage, and mechanistic studies on drug delivery agents and pathways. Here, we outline case studies where spontaneous Raman microscopy and SRS microscopy have advanced current understanding in each of these areas. Clearly, the benefits provided by Raman imaging are not restricted to biomedical applications, and thus the final section highlights recent advances in the Raman imaging of agrochemical uptake.

### 4.1 Case study 1: cellular drug distribution

Growing evidence suggests that measurement of the physiochemical properties of a drug molecule within early stage drug discovery and development is crucial to reducing the current high attrition rates of new chemical entities on entering late stage development programmes,^82^ and an improved understanding of the cellular concentration and localisation of a new drug molecule in relevant cells and tissues, represents a key challenge in early stages of drug discovery campaigns.^83^ Specifically, poor biopharmaceutical properties associated with drug uptake, retention, metabolism and solubility are directly related to poor clinical success.^84,85^ The application of imaging modalities to drug discovery programmes has the potential to accelerate preclinical lead optimisation cycles and enhance the *in vitro* to *in vivo* translation of drug candidates.^11,16^

**Fig. 14:**
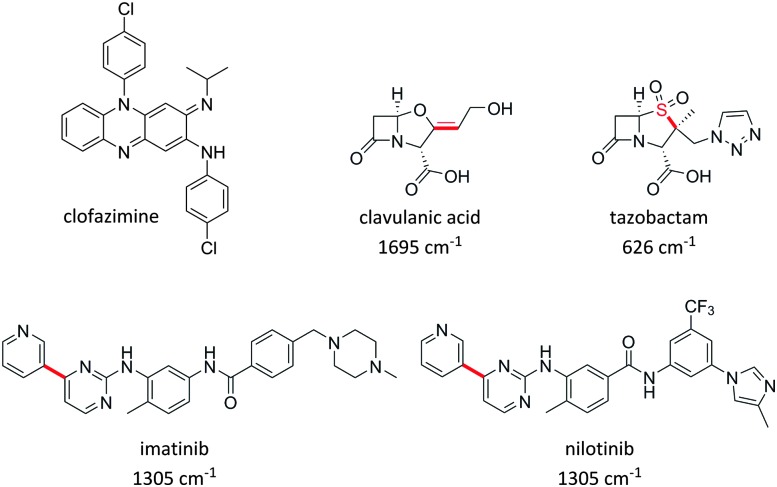
Structures of drugs which have been imaged by spontaneous Raman spectroscopy, and SRS microscopy, highlighting the key bonds and Raman stretching frequencies which have enabled their intracellular quantitation. Note: for clofazimine a range of peaks were used in the region 1100–1600 cm^–1^.

Several examples of studies which use Raman imaging to probe intracellular drug distribution have been published very recently. Firstly, Baik *et al.* used spontaneous Raman spectroscopy to monitor the intracellular accumulation of the antibiotic clofazimine (Fig. 14).^22^ This study also confirmed the amorphous nature of the drug in the discrete clofazimine inclusions formed in the cytoplasm by comparison of their Raman spectra with amorphous and crystalline forms of the solid drug. Carey *et al.* monitored the penetration of two antibiotics, clavulanic acid and tazobactam, into bacterial cells based upon the Raman activity of characteristic features from clavulanic acid (1695 cm^–1^ C=C band) and tazobactam (626 cm^–1^ C–S stretch) (Fig. 14).^23^ Changes in the spontaneous Raman spectrum of these drug molecules during a drug treatment cycle were monitored, and the formation of the acyl–enzyme complex between the inhibitors and the β-lactamase enzyme could also be detected. However, the protocol employed on the drug-treated cells for visualisation by spontaneous Raman required lengthy pelleting and freeze-drying procedures, prohibiting direct live-cell imaging and drug localisation studies.

Erlotinib is a clinically relevant drug, which is used in the treatment of non-small cell lung cancers, and has been recently studied by confocal Raman microscopy, using the inherent alkyne bond as a marker for intracellular visualisation (see Fig. 10).^49^

**Fig. 15:**
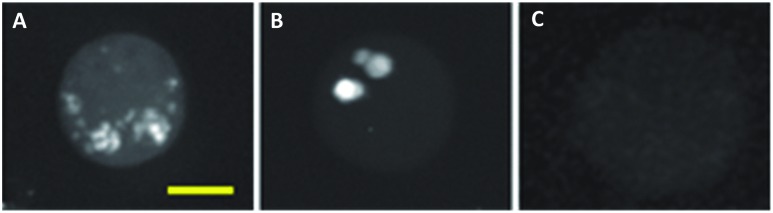
Maximum-intensity projection of 3D SRS images at 1305 cm^–1^ of BaF3/BCR-ABL1 cells incubated with (A) imatinib (20 μM, 4 h), (B) nilotinib (20 μM, 4 h), and (C) DMSO only. Scale bar 5 μM. Adapted from ref. 86 with permission. Copyright (2014) Nature Publishing Group.

More recently, advances in hsSRS imaging have enabled the intracellular visualisation and localisation of other un-modified drugs which do not contain a bioorthogonal functional group. Fu *et al.* elegantly described the first direct observation and quantification of tyrosine kinase inhibitors (TKIs) in living chronic myelogenous leukaemia (CML) cells.^86^ Prior acquisition of the spontaneous Raman spectrum for each of the drugs enabled identification of their major Raman bands. Cellular imaging of these drugs by hsSRS used peaks that were inherent to the structure of the drug compounds and were not necessarily located within the ‘cellular silent region’; potentially widening the scope of this approach over spectroscopically bioorthogonal tagging strategies. Fig. 15 shows the SRS images of BaF3/BCR-ABL1 cells treated with imatinib, nilotinib and DMSO (control) obtained in this study. Lysosomal localisation of these drugs was corroborated by co-localisation with the fluorescent stain LysoTracker Red, using two-photon excitation. Since SRS signals show a linear dependence on concentration, intracellular drug concentrations can be determined based on calibrations against standard solutions of each of the drugs. Cells were treated with 20 μM imatinib or 20 μM nilotinib, and imaged by hsSRS. Both drugs were enriched by more than 1000-fold in lysosomes; hence reducing the availability of cytosolic drug, and ultimately reducing drug efficacy. Analysis of the hsSRS images also showed the cytosolic concentrations of these drugs to be below the current detection limit (1–2 mM). This study shows that hsSRS imaging offers unprecedented capability in label-free drug visualisation, and is applicable to pharmacokinetic studies.

Liposomal drug carriers can also be imaged *via* Raman microscopy. Chernenko *et al.* incubated MCF-7 cells with a deuteriated liposomal drug carrier (DSPC-*d70*), to determine the distribution patterns of these drug delivery systems.^47,87^ When images were acquired using the C–D stretching region between 2000–2310 cm^–1^, a cytoplasmic distribution of DSPC-*d70* and its accumulation in the cell periphery were observed. However, the spontaneous Raman microscope used in these studies required lengthy scanning times (approximately 1 h required to image one entire cell), thus illustrating the clear need for the video-rate image acquisition achieved with SRS microscopy. Other drug delivery mechanisms analysed by spontaneous Raman spectroscopy include targeted drug delivery *via* gold^88,89^ and graphene oxide silica nanoparticles,^90^ biodegradable polymeric nano-carrier systems^91^ and nanocolloids.^92^

### 4.2 Case study 2: cellular metabolism and lipid storage

Maintaining physiological homeostasis requires strict regulation of a multitude of cellular processes. The control of cellular and systemic lipid levels is of particular significance in maintaining a healthy phenotype, with excessive cholesterol levels resulting in toxicity, and dysregulation of sterol metabolism being a hallmark of diabetes and atherosclerosis.^93^ Imbalances in lipid metabolism have been implicated in several diseases (including cancer, atherosclerosis, and diabetes), highlighting the importance of understanding lipid biochemistry in disease.^94,95^ Thus mapping lipid oxidation, the degree of lipid unsaturation and cholesterol storage *in vivo* may prove fruitful in developing novel approaches to tackling a range of diseases. To this end, several recent studies have demonstrated Raman imaging of lipid storage and metabolism.

**Fig. 16:**
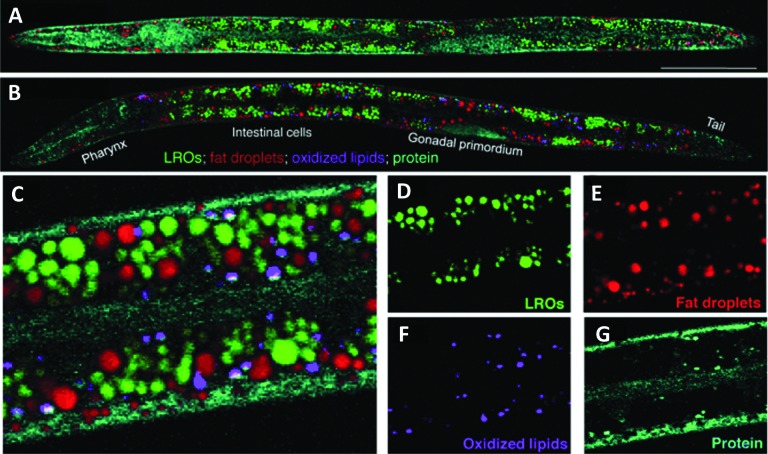
Compositional analysis of intracellular compartments in whole *C. elegans* worms by hsSRS imaging, k-means clustering, and MCR analysis. (A and B) MCR-retrieved concentration maps of neutral fat droplets, lysosome-related organelles (LROs), oxidised lipids, and protein in the body of whole wild type worms (A) and daf-2 mutants (B). Scale bar: 50 μm. (C) Zoom-in of intestine cells indicated in (B). (D–G) MCR-reconstructed concentration images of LROs, fat droplets, oxidised lipids, and protein, respectively. Adapted from ref. 97 with permission. Copyright (2014) Wiley-VCH Verlag GmbH & Co kGaA, Weinheim.

*C. elegans* can provide useful predictive data for studying drug-target interactions and for target validation.^96^ hsSRS was used by Wang *et al.* for *in situ* identification and quantification of distinct lipid compartments in living *C. elegans* (Fig. 16).^97^ Imaging in the region 1620–1800 cm^–1^ [which covers (i) sterol C=C (1669 cm^–1^), (ii) triglyceride C=C (1655 cm^–1^) and C=O (1745 cm^–1^), and (iii) protein amide I C=O (1650 cm^–1^) bands] enabled the generation of quantitative chemical maps based upon the intensity ratio of the Raman bands, and has allowed the analysis of lipid unsaturation, lipid oxidation and cholesterol storage, through multivariate curve analysis (MCR). These data showed that lysosome related organelles (LROs) are cholesterol storage sites in *C. elegans.* Similar approaches have been used to image cholesterol storage in atherosclerotic arterial tissues.^98^

hsSRS has also been used by Fu and co-workers for the quantification of two major lipidic components in cells and tissues, cholesteryl ester and triacylglycerol.^99^ Their approach highlighted previously unknown changes in lipid composition associated with obesity and related diseases.^99^ These direct measurements of the impact of lipid storage and biochemistry, further demonstrate the applicability of SRS imaging *in vivo* as a tool for the drug discovery process.

### 4.3 Case study 3: dermal drug delivery

Diseases of the skin are typically alleviated by application of topical drug formulations. Dermal formulations can offer advantages over oral drug delivery because direct targeting of the local affected area is easily achieved through dermal application, compared to systemic exposure through oral delivery. However, the impermeable nature of skin hinders the widespread uptake of chemical species, with only small lipophilic compounds effectively delivered *via* the transdermal route.^100^ Recent reports indicate that despite significant research efforts focussed upon transdermal drug discovery, only modest achievements have been realised.^101^

Excised specimens of animal and human skin have been used as models to test drug permeation. However, practical problems may arise with a lack of available tissue and the quality of excised tissue. Furthermore, cutaneous bioavailability studies are often performed using adhesive tape stripping, whereby the outermost layer of the skin, the stratum corneum (SC), is sequentially revealed, and the concentration of drug present is determined with each strip, to produce a concentration profile across the tissue depth.^102^ This approach is hampered by issues relating to the timing of the tape strippings, the number of removals which can make the process onerous and unsuitable for high throughput screening, and whether the strips reveal the same amount of SC each time. Therefore, alternative approaches for monitoring the bioavailability of drugs and their excipients would be beneficial to drug discovery programmes focussed upon dermal delivery.

Raman spectroscopy is emerging as an alternative approach to understanding topical drug delivery;^102,103^ and SRS may potentially enable the analysis of drug toxicity on live skin, which is not feasible using tape stripping on excised specimens. SRS microscopy has been used to probe epidermal architecture by collecting images at frequencies corresponding to protein (2950 cm^–1^), lipid (2850 cm^–1^) and water (3340 cm^–1^). Depth profiles were collected to a range of 28 μm, to provide further insight into the chemical nature of micro-anatomical features of skin.^103^ A deuteriated oleic acid, was shown to distribute to lipid rich regions within the skin after topical application.^103^ This allowed the differentiation of drug localisation, which could not be determined by the tape stripping methods.

**Fig. 17:**
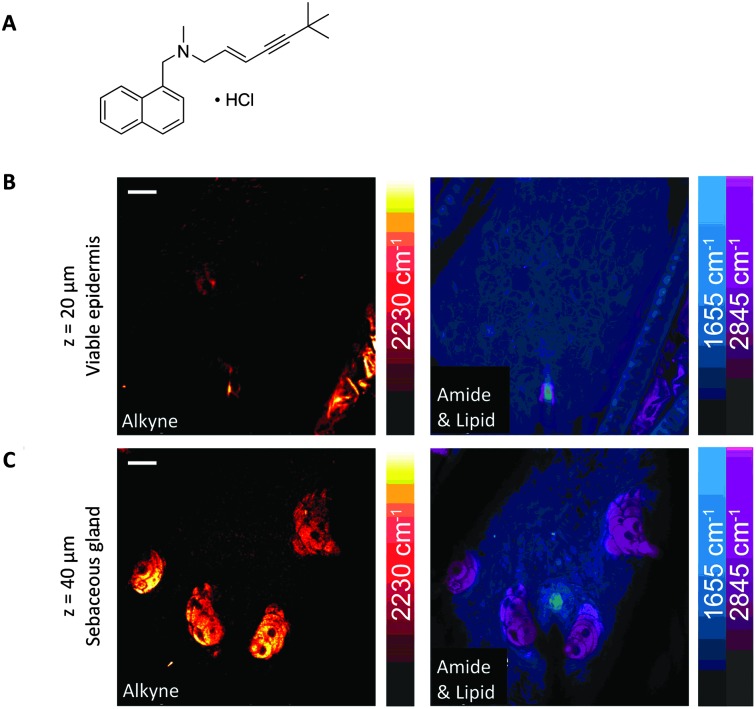
SRS imaging of mouse ear skin with topically applied terbinafine hydrochloride (TH). (A) Terbinafine hydrochloride. (B) SRS imaging at the viable epidermis layer (*z* = 20 μm), (C) SRS imaging at the sebaceous gland layer (*z* = 40 μm) of mouse ear skin. In both (B and C), composite images show both protein (1655 cm^–1^) and lipid (2845 cm^–1^) distributions, and the alkyne images indicate that the TH penetrates *via* the lipidic phase. Scale bars, 20 μm. Adapted from ref. 60 with permission. Copyright (2014) Nature Publishing Group.

Drug delivery to the skin has also been demonstrated through monitoring the prominent Raman bands of DMSO and retinoic acid, and mapping their cell penetration through depth profiling SRS microscopy.^27^ Lateral profiling of topically applied retinoic acid (1570 cm^–1^) and DMSO (670 cm^–1^), demonstrated that the hydrophobic retinoic acid penetrates through the lipid rich regions, whilst the hydrophilic DSMO avoids lipid-rich skin structures such as adipocytes in the subcutaneous fat layers.^27^ SRS microscopy has also demonstrated that the alkyne-containing anti-fungal dermal drug, terbinafine hydrochloride (TH), penetrates through skin in the lipid phase (Fig. 17).^60^ Finally, the uptake of a range of pharmaceutically relevant solvents (water, propylene glycol and DMSO) in human nail samples has also been imaged by SRS using their deuteriated isotopologues to provide label-free chemical contrast.^104^

Optimisation of topical drug delivery for the treatment of several dermatological diseases was explored by Xie and Guy, who demonstrated the capability of SRS to provide kinetic and mechanistic insights into the transdermal drug delivery process.^105^ Notably, SRS imaging provided evidence of drug precipitation from the applied formulation, post application onto the skin. Whilst following the penetration of deuteriated ibuprofen in un-deuteriated propylene glycol, images of the C–D stretching frequency (2120 cm^–1^) of the ibuprofen-*d*_3_ confirmed the formation of ibuprofen crystals at the skin surface. Belsey *et al.* also demonstrated time course imaging of porcine skin dosed with ketoprofen in deuteriated propylene glycol by SRS imaging.^106^

In summary, SRS microscopy has advanced current understanding within dermato-pharmacokinetics, highlighting previously unknown aspects of drug delivery to the skin. Novel features of drug delivery have been identified including the different rates of drug penetration *via* hair follicles and through intracellular diffusion, and interesting features of formulation application. Monitoring changes in the morphology of skin cells *via* SRS imaging following drug application, may enable toxicity studies to be performed on topically applied drugs, significantly improving on available methodologies.

### 4.4 Case study 4: agrochemical uptake

The development of agrochemical agents including herbicides, fungicides and insecticides relies heavily on understanding the environmental impact the agrochemicals and any potential metabolites will have. Furthermore, prior to achieving regulatory approval, studies to demonstrate that the compounds do not persist in the environment considerably beyond their period of intended use, and that they are efficacious against their intended target are required.^107,108^ Such information is necessary because typically agrochemicals are applied over large areas in agricultural and urban environments.^108^ Importantly, understanding the uptake, translocation and metabolism of herbicides into foliar tissue and roots directly influences the activity, persistence and crop selectivity in the field. Traditional methods of measuring uptake and movement of agrochemicals include HPLC, autoradiography and liquid scintillation techniques.^109^ Recently, Clench *et al.* have demonstrated a mass spectrometry based imaging approach to following agrochemical translocation in sunflower plants.^110^ Such techniques however, are limited by factors such as their destructive nature (making it difficult to obtain information from living plant leaves) and the time and expense of radiochemical synthesis (prohibiting uptake studies in the early stage agrochemical development).

**Fig. 18:**
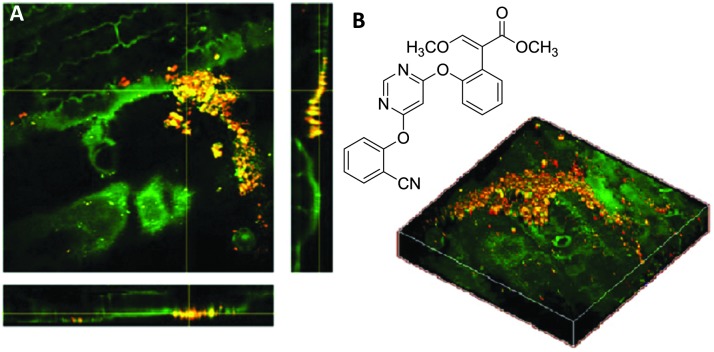
Application of azoxystrobin to maize leaves. (A) Azoxystrobin applied to a maize leaf (red = 2225 cm^–1^ from the C≡N bond, green = 2930 cm^–1^ from the CH_3_ vibrations) to show crystalline deposits of the chemical on the leaf surface, which appear yellow/orange due to contrast from the CH bonds (green) and CN bonds (red) which have been combined. (B) Chemical structure of azoxystrobin. Adapted from ref. 112 with permission. Copyright (2013) American Chemical Society.

Conventional Raman microscopy has been utilised to study the chemical composition of various features of plant leaves.^111^ More recently, Mansfield *et al.* demonstrated the applicability of SRS imaging for the analysis of plant cell wall components, epicuticular waxes and the deposition of agrochemical formulations directly onto leaf surfaces.^112^ Interestingly, the group were able to use the nitrile functional group present within two agrochemicals, chlorothalonil and azoxystrobin to perform spectroscopically bioorthogonal SRS microscopy (at 2234 and 2225 cm^–1^ respectively), which revealed crystalline deposits on the leaf surfaces (Fig. 18), whilst the CH_3_ vibrations at 2930 cm^–1^ provided structural information on the plant cell walls.

Finally, SRS microscopy has been extended to the imaging of perfluorinated agrochemicals, which was achieved *via* excitation of the C–F bonds at 687.5 cm^–1^.^52^ This use of a vibrational frequency which lies outside the cell silent region provides an important expansion to the current ‘toolbox’ approach to chemical labelling; with a functional group which is both medicinally relevant, and offers significant benefits for SRS detection. These early studies represent novel approaches to understanding agrochemical uptake in plants in a rapid, non-destructive manner, which could therefore present new opportunities in early stage agrochemical development.

## 5. Conclusions

Live cell Raman imaging when coupled with bioorthogonal labelling strategies is emerging as a promising imaging modality applicable to drug discovery and medicine. The direct visualization of bioorthogonal Raman reporters avoids the need for further fixing, and click-labelling strategies, which thereby improves sample throughput and accommodates live-cell imaging. The low sensitivity of the Raman scattering process still remains a significant limitation of the approach, where in many cases, typical detection limits are above physiologically relevant drug concentrations. Advances in Raman tag design and technical instrument improvements are expected to make further gains in detection sensitivity. The use of advanced Raman scattering techniques, namely SRS, and the incorporation of hyperspectral and multiplexing approaches will also likely transcend detection and imaging speed limits. However, these early studies indicate the exciting potential of Raman microscopies to shed light on our understanding of how small molecules interact with cells and tissues, providing significant benefits to early-stage drug discovery projects.
